# Maternal Near Miss and quality of care in a rural Rwandan hospital

**DOI:** 10.1186/s12884-016-1119-1

**Published:** 2016-10-21

**Authors:** Richard Kalisa, Stephen Rulisa, Thomas van den Akker, Jos van Roosmalen

**Affiliations:** 1 Department of Obstetrics and Gynecology, Ruhengeri Hospital, Musanze, Rwanda; 2 Athena Institute, VU University, Amsterdam, The Netherlands; 3 Department of Obstetrics and Gynecology, University of Rwanda, Kigali, Rwanda; 4 Department of Obstetrics, Leiden University Medical Centre, Leiden, The Netherlands

**Keywords:** Audit, Maternal morbidity, Maternal mortality, Maternal near miss, Obstetrics, Quality of care

## Abstract

**Background:**

The WHO Maternal Near Miss (MNM) approach was developed to evaluate and improve quality of obstetric care worldwide. This study aimed to study the incidence of MNM and quality of care at a district hospital in rural Rwanda by applying this approach.

**Methods:**

A facility based, prospective cohort study conducted at a district hospital in rural Rwanda between June 2013 and December 2014. Subjects were followed from time of admission to discharge or death.

**Results:**

In 3979 deliveries, 3827 singletons and 152 twins pairs were born. Among the 4131 neonates, there were 3994 live births and 137 stillbirths. Ninety-nine women suffered severe maternal outcome (SMO): 86 maternal near misses and 13 deaths. This adds up to a maternal near miss ratio of 21.5 per 1000 live births (95 % CI 17.3-26.5), a maternal mortality ratio of 325 per 100 000 live births (95 % CI 181–543) and a mortality index of 13.1 % (95 % CI 7.3–21.9). Hemorrhage (*n* = 49, 57 %) and hypertensive disorders (*n* = 27, 31.4 %) were the commonest MNM conditions. Eclampsia (*n* = 4/13; 30.7 %) was the leading cause of maternal mortality, while sepsis/peritonitis following cesarean section (*n* = 2/6; 33.3 %) had the highest mortality index. Seventy-seven out of 99 SMO cases (77.9 %) were referred from other facilities with critical conditions and 28 out of 99 SMO cases (28.3 %) were admitted into the Intensive Care Unit. Several indicators such as administration of oxytocin, magnesium sulfate and antibiotics were found to be suboptimal.

**Conclusions:**

MNM is common at district level in Rwanda. The MNM approach enabled us to identify shortfalls in clinical practice and the referral system.

## Background

Most maternal deaths occur during the intrapartum and immediate postpartum period from preventable or treatable causes such as hemorrhage, eclampsia and sepsis [1]. The majority of these women die in low-income countries, and several countries in sub-Saharan Africa have very high maternal mortality ratios (MMR), often double the estimated global MMR of 400 per 100,000 live births [2].

Although global and regional population based numbers of maternal deaths remain high, numbers at the facility level are relatively low. It has been shown that reviewing critical incidents, including obstetric audits at facility level, may improve quality of facility based care [3] and functioning of the broader health system [4–6].

Until recently, a variety of criteria was used to define severe maternal morbidity or maternal near miss (MNM) [4]. In 2011, the World Health Organization (WHO) developed a systematic MNM approach. The aim was to facilitate comparisons between different studies, and between countries and regions [7], in order to address shortcomings in quality of obstetric care and improve pregnancy outcome [8]. Within this approach, an MNM refers to ‘a woman who almost died but survived a complication during pregnancy, childbirth, or within 42 days after termination of pregnancy’ [9, 10], and organ failure based criteria reflect critical illness and allow for identification of MNM. A number of studies have applied these criteria in low-income countries [8, 11–14].

In Rwanda, the MMR has been reduced by roughly two-thirds from 750 in 2005 to 210 per 100 000 live births in 2015 [15, 16]. This may have resulted from strong government commitment combined with a well-organized community based health program, performance based financing, innovative community health insurance, and SMS-based alert systems among other policy changes [17, 18]. Nevertheless, additional progress is needed, and evaluation of MNM may be of increasing use in light of this mortality reduction. This study aimed to assess MNM characteristics and process indicators related to quality of care at a district hospital in rural Rwanda, by applying the WHO MNM approach.

## Methods

This was a prospective cohort study among pregnant women who were admitted for delivery or pregnancy related complications, and who sustained severe acute maternal morbidity at Ruhengeri maternity ward in Musanze district, Rwanda, between June 2013 and December 2014. Musanze is located in northern Rwanda, at 94 km from the capital Kigali, and has a population of 368 267 inhabitants with a total fertility rate of 4.6 births per woman [16].

Ruhengeri hospital acts as a provincial referral hospital for high-risk obstetric cases from health centers and other district hospitals in the northern province. Annually, 3500 deliveries are conducted at the facility. During the study period, medical staff consisted of one specialist obstetrician, four medical officers, two intern doctors and 18 midwives. Patients with critical conditions were admitted into the intensive care unit (ICU), where vital signs were monitored hourly, and treatment with vasoactive drugs and mechanical ventilation could be provided. Blood for transfusion was supplied by the regional blood bank located next to the hospital.

Eligibility for the study was not restricted by gestational age. Therefore, women who had had an abortion or ectopic pregnancy and met the WHO MNM criteria were also included [7, 10]. Women who developed complications more than 42 days after termination of pregnancy were not eligible. On a daily basis, we captured relevant data for every woman who presented with severe acute maternal morbidity or died during admission, by using available medical records. Severe Maternal Outcome (SMO) was defined as MNMs and maternal deaths combined.

Two trained research assistants screened possible participants and the principal investigator (RK) verified suitability for study inclusion. Possible participants included patients who had severe maternal complications at baseline, received critical interventions or were admitted into the ICU, according to WHO guidelines [10]. We tried as much as possible to apply the WHO MNM criteria but had to make several modifications due to lack of laboratory tests and management options (Table 1). For every case, information was collected regarding socio-demographic characteristics, gestational age, maternal and neonatal outcome, as well as process indicators regarding prevention and treatment of the main causes of MNM and death: giving magnesium sulfate for treatment of eclampsia, oxytocin for prevention and treatment of postpartum hemorrhage, and antibiotics for prophylaxis (during cesarean section) and treatment of sepsis.

**Table 1:** WHO near miss criteria and modifications for Ruhengeri hospital

WHO near miss criteria [10]	Ruhengeri near miss criteria
Clinical Criteria	
Acute Cyanosis	Not modified
Gasping	Not modified
Respiratory rate > 40 or < 6/min	Not modified
Shock^a^	Not modified
Oliguria non responsive to fluids or diuretics^b^	Not modified
Failure to form clots^c^	Not modified
Loss of consciousness lasting > 12 h^d^	Not modified
Cardiac arrest^e^	Not modified
Stroke^f^	Not modified
Uncontrollable fit/total paralysis^g^	Not modified
Jaundice in the presence of pre-eclampsia^h^	Not modified^i^
Laboratory-based criteria	
Oxygen saturation < 90 % for ≥ 60 min	Not modified
PaO2/FiO2, 200 mmHg	Not available^j^
Creatinine ≥300 mmol/l or ≥ 3.5 mg/dL	Not modified
Bilirubin > 100 mmol/l or .6.0 mg/dL	Not modified
pH < 7.1	Not available
Lactate > 5 mEq/mL	Not available
Acute thrombocytopenia (< 50,000 platelets/ml)	Not modified
ketoacids in urine	Not available
Management-based criteria	
Admission to intensive care unit	Not modified
Use of continuous vasoactive drugs	Not modified
Hysterectomy following infection or hemorrhage	Not modified
Transfusion of ≥ 5 units of blood	Not modified
Intubation and ventilation for ≥ 60 min not related to anesthesia	Not modified
Dialysis for acute renal failure	Not available
Cardio-pulmonary resuscitation	Not modified
Severe maternal complications	
	Eclampsia^k^
	Sepsis or severe systemic infection^l^
	Ruptured uterus^m^

^a^Shock is defined as a persistent severe hypotension, defined as a systolic blood pressure < 90 mmHg for 60 min with a pulse rate of ≥ 120/min despite aggressive fluid replacement (> 2 L)

^b^Oliguria is defined as an urinary output < 30 ml/h for 4 h or < 400 ml/24 h

^c^Failure to form clots is defined as the absence of clotting from the IV site after 7–10 min

^d^Unconsciousness/coma lasting > 12 h is defined as a profound alteration of mental state that involves complete or near-complete lack of responsiveness to external stimuli or Glasgow Coma Scale < 10

^e^Cardiac arrest is defined as loss of consciousness and absence of pulse or heart beat

^f^Stroke is defined as a neurological deficit of cerebrovascular cause that persists ≥ 24 h, or is interrupted by death within 24 h

^g^Uncontrollable fit is a condition in which the brain is in state of continuous seizure

^h^Pre-eclampsia: the presence of hypertension associated with proteinuria. Hypertension is defined as a blood pressure ≥ 140 mmHg (systolic) or ≥ 90 mmHg (diastolic). Proteinuria is defined as excretion of ≥ 300 mg protein/24 h or 300 mg protein/l urine or ≥ 1+ on a dipstick

^i^Was not regularly available due to lack of resources

^j^Not available

^k^Eclampsia is defined as the presence of hypertension associated with proteinuria and fits

^l^Sepsis is defined as a clinical sign of infection and 3 of the following: temp > 38C or < 36C, respiration rate > 20/min, pulse rate > 90/min, WBC > 12, clinical signs of peritonitis

^m^Uterine rupture is defined as the complete rupture of a uterus during labor

Data were entered into Microsoft Excel, and subsequently entered into STATA version 12 for analysis. Outcomes were given in proportions with 95 % confidence intervals (CIs). The results are presented in accordance with the WHO MNM approach.

## Results

During the 19-month study period, there were 3979 deliveries, in which 3827 singletons and 152 twins pairs were born. Among the 4131 neonates, there were 3994 live births, and 137 stillbirths. Three hundred thirty two women sustained potentially life-threatening conditions and 99 had SMO: 86 near misses and 13 maternal deaths. The cesarean section rate for Ruhengeri hospital was 34.9 %.

Cases of morbidity were classified by underlying causes and type of organ system dysfunction (Tables 2 and 3). The commonest conditions contributing to MNM were severe hemorrhage (*n* = 49; 57 %), but with MI 5.8 % (95 % CI 1.5–15.7). Severe hypertensive disorders was the second main cause of morbidity (*n* = 27; 31.4 %) with MI 13 % (95 % CI 4.1–31.1). Eclampsia (*n* = 4/13; 30.7 %) was the leading cause of maternal mortality, while sepsis/peritonitis following cesarean section (*n* = 2/6; 33.3 %) had the highest mortality index. Other common contributing factors among MNM cases were lack of health insurance (*n* = 23; 26.7 %), anemia (*n* = 21; 24.4 %) and previous cesarean section (*n* = 12; 14 %).

**Table 2:** Underlying causes and contributory factors of potentially life-threatening conditions (PLTC) and severe maternal outcomes^a^

	Women with PLTC	MNM	Maternal deaths	MNMR/1000 live birth	Mortality index
	332 (%)	86 (%)	13 (%)		(%)
Underlying causes^b^					
Severe hemorrhage	134 (40.4)	49 (57)	3 (23.1)	12.3 (9.2–16.1)	5.8 (1.5–15.7)
Abortion	33 (9.9)	7 (8.1)	0 (0)		
Ectopic	21 (6.3)	13 (15.2)	0 (0)		
Molar	4 (1.2)	0 (0)	0 (0)		
Placental abruption	10 (3.0)	4 (4.6)	0 (0)		
Placenta Previa	17 (5.1)	6 (7.0)	0 (0)		
Postpartum Hemorrhage	49 (14.8)	19 (22.1)	3 (23.1)		
Hypertensive disorders	93 (28.0)	27 (31.4)	4 (30.7)	6.8 (4.6–9.7)	13 (4.1–31.1)
Severe Pre-eclampsia	40 (43.0)	7 (8.1)	0 (0)	–	
Eclampsia	53 (57.0)	20 (23.3)	4 (30.7)		
Pregnancy related infections^c^	40 (12.1)	0 (0)	2 (15.4)	–	
Sepsis/peritonitis post-cesarean	38 (11.4)	4 (4.7)	2 (15.4)	1.0 (0.3–2.4)	33.3 (5.6–110.1)
Unanticipated complications of management^d^	0 (0)	2 (2.3)	1 (7.7)	0.5 (0.1–1.7)	14.3 (1.6–164.4)
Coincidental conditions^e^	2 (0.6)	0 (0)	0 (0.0)	–	
Unknown	0 (0)	0 (0)	1 (7.7)	–	
Other obstetric complications^f^	16 (4.8)	4 (4.7)		–	
Associated conditions					
Anemia	73 (22)	21 (24.4)	4 (30.8)	5.3 (3.3–7.9)	16.0 (5.1–38.6)
HIV	16 (4.8)	8 (9.3)	2 (15.4)	2.0 (0.9–3.8)	20.0 (3.3–66.1)
Previous CS	22 (6.6)	12 (14)	1 (7.7)	3.0 (1.6–5.1)	7.7 (0.3–37.9)
Prolonged/obstructed Labor	5 (1.5)	5 (5.8)	1 (7.7)	1.3 (0.5–2.8)	16.7 (0.8–82.2)
Lack of medical Insurance	28 (8.4)	23 (26.7)	4 (30.8)	5.8 (3.7–8.5)	14.8 (4.7–35.7)

^a^Values given as number (percentages)

^b^One woman could have experienced more than one cause

^c^Pyelonephritis (12 PLTC), Hyperemesis (10 PLTC), Choriamnionitis (8 PLTC), Endometritis (8 PLTC), severe malaria (2 PLTC, 1 MD), HIV encephalopathy (1 MD)

^d^Iatrogenic complications (2 MNM, 1 MD)

^e^Road traffic accident, Stevens-Johnson syndrome (2 PLTC)

^f^Asthmatic crisis (6 PLTC), Herbal intoxication (4 PLTC), Epilepsy (3 PLTC, 1 MNM), Unknown cause of Jaundice (2 PLTC), Blood transfusion reaction (1 PLTC, 1MNM), Peripartum cardiomyopathy (2 MNM)

**Table 3:** Frequencies of maternal near miss and maternal death by type of organ system dysfunction

Organ dysfunctions	MNM cases *n* = 86 (%)	Maternal death *n* = 13 (%)	MNM mortality ratio	Mortality index (%) 95 % CI
Cardiovascular	56 (65.1)	7 (53.8)	8 :1	11.1 (4.9–21.)
Renal	5 (5.8)	1 (7.7)	5 :1	16.7 (0.8–82.2)
Respiratory	25 (29.1)	6 (46.2)	4 :1	19.4 (7.9–40.3)
Hepatic	6 (7.0)	3 (23.1)	2 :1	33.3 (8.5–90.7)
Neurologic	26 (30.2)	5 (38.5)	5 :1	16.1 (5.9–35.8)
Hematologic	16 (18.6)	7 (53.8)	2 :1	30.4 (13.3–60.2)
Hysterectomy/Uterine	8 (9.3)	3 (23.1)	3 :1	27.3 (6.9–74.2)
Multiple organ	35 (40.7)	9 (69.2)	4 :1	20.5 (9.9–37.5)
Unspecified organ	–	2 (15.4)		–

Most common organ dysfunctions among women with MNM conditions were cardiovascular dysfunction (*n* = 56; 65.1 %) followed by multiple organ dysfunction (*n* = 35; 40.7 %). The mortality index was highest for hepatic dysfunction, (33.3 %). The MNM/mortality ratio was highest for cardiovascular dysfunction (8:1), followed by renal and neurologic dysfunction (5:1) (Table 3).

Cesarean section was performed in many women with potentially life threatening conditions (*n* = 125/332; 37.7 %), MNM (*n* = 37/86; 43 %) and maternal deaths (*n* = 3/13; 23.1 %). Neonatal outcomes (preterm births, stillbirths and perinatal deaths) were worst among maternal deaths and comparable between women with potentially life threatening conditions and maternal near misses (Table 4).

**Table 4:** End of pregnancy and birth outcomes in our study population

	No severe complication (%)	Potentially life-threatening conditions (%)	Maternal near miss cases (%)	Maternal deaths (%)	All women
	3922	332	86	13	4353
End of pregnancy					
Vaginal Delivery	2344 (59.8)	113 (34.1)	18 (20.9)	7 (53.8)	2482 (57.1)
Vacuum Extraction	156 (3.9)	37 (11.1)	0 (0.0)	0 (0.0)	193 (4.4)
Cesarean section	1277 (32.6)	125 (37.7)	37 (43)	3 (23.1)	1442 (33.1)
Complete abortion, vacuum aspiration/curettage	94 (2.4)	33 (9.9)	7 (8.1)	0 (0.0)	134 (3.1)
Laparotomy for ruptured uterus	0 (0.0)	3 (0.9)	11 (12.8)	1 (7.7)	15 (0.3)
Women discharged or died still pregnant	17 (0.4)	0 (0.0)	0 (0.0)	2 (15.4)	19 (0.4)
Laparotomy for ectopic pregnancy	34 (0.9)	21 (6.3)	13 (15.2)	0 (0.0)	68 (1.6)
Cesarean section rate^a^	n/a (33.8)	n/a (45)	n/a (56.1)	n/a (27.3)	n/a (34.9)
Preterm Birth	268 (7.1)	32 (11.5)	5 (7.6)	2 (18.1)	307 (7.4)
Fresh Stillbirth	46 (1.2)	5 (1.8)	6 (9.1)	3 (27.3)	60 (1.5)
Macerated Stillbirth	76 (2.0)	0 (0.0)	0 (0.0)	1 (9.1)	77 (1.9)
Perinatal death^b^	107 (2.83)	17 (6.1)	6 (9.1)	5 (45.5)	130 (3.1)

^a^Cesarean deliveries divided by all deliveries/^b^Fetal deaths + intra hospital early neonatal mortality

The SMO ratio was 24.8 per 1000 live births (95 % CI 20.3–30.1), and the MNM ratio 21.5 per 1000 live births (95 % CI 17.3–26.5). The maternal mortality ratio (MMR) was 325 (95 % CI 181–543) per 100 000 live births, the mortality index (MI = maternal deaths/MNM+ maternal deaths) 13.1 % (95 % CI 7.3–21.9), and the maternal near miss mortality ratio (MNM: maternal deaths) 7:1 (Table 5). In 332 women, 234 critical interventions were carried out, including 108 blood transfusions (32.5 % of women) and 71 laparotomies (21.4 % of women). The ICU admission rate was 1.4 % (61/4353) among all women, whereas ICU admission rate among women with SMO was 28.3 % (28/99) and proportion of maternal deaths occurred without ICU admission was 46 % (6/13) (Table 5). Of 134 women with severe hemorrhage, 52 (38,8 %) were transfused five or more units of blood.

**Table 5:** Severe maternal outcomes, near miss indicators and facility-related indicators, including 95 % confidence intervals

Maternal outcomes and indicators^a^	Value
Severe maternal outcomes (SMO) cases (*n*)	99
Cases of maternal death (*n*)	13
Cases of maternal near miss (*n*)	86
Overall near miss indicators	
^c^Severe maternal outcome ratio (per 1000 live births)	24.8 (20.3–30.1)^b^
Maternal near miss incidence ratio (per 1000 live births)	21.5 (17.3–26.5)^b^
^d^Maternal near miss mortality ratio	7:1
^e^Mortality index	13.1 % (7.3–21.9)
Hospital access indicators^b^	
SMO cases presenting with organ dysfunction or maternal death within 12 h of hospital stay (SM012) (*n*)	79
Percentage of SM012 cases among all SMO cases	79.8 (63.6–98.9)
Percentage of SM012 cases coming from other health facilities	77.8 (61.8–96.7)
SM012 mortality index	8.1 (3.6–16.1)
Intra-hospital care	
Intra-hospital SMO cases of organ dysfunction or maternal death after 12 h of hospital stay	32
Rate of intra-hospital SMO (per 1000 live births)	8.0 (5.6–11.2)
Intra-hospital mortality index	15.8 (6.4–32.8)
Intensive care used^a^	
Percentage of ICU admission	1.4 (1.1–1.8)
Percentage of ICU admission among women with SMO	28.3 (19.2–40.3)
Percentage of SMO among women admitted to ICU	73.8 (54.5–97.8)
Percentage of maternal deaths occurred without ICU admission	46.2 (18.7–96)

*Abbreviations*: *ICU* intensive care unit or admission also included women who stayed in the recovery room on the labor ward for longer than 6 h, *SMO* severe maternal outcome

^a^Based on data from all women (*n* = 4353)

^b^The denominator for these 2 indicators is ‘live births’ (*n* = 3994)

^c^ *SMOR* Severe Maternal Outcome Ratio: the number of women with life-threatening conditions (MNM+ MD) per 1000 live births (LB). This indicator gives an estimate of the amount of care and resources that would be needed in an area or facility (SMOR = MNM + MD/LB) [10]

^d^ *MNMR* Maternal near miss mortality ratio: the number of maternal near miss cases per 1000 live births (MNMR = MNM/LB). Similarly to the SMOR, this indicator gives an estimation of the amount of care and resources that would be needed in an area or facility [10]

^e^Mortality Index (MI): the number of maternal deaths divided by the number of women with life-threatening conditions expressed as a percentage (MI = MD/MNM + MD). The higher the index the more women with life-threatening conditions die (low quality of care), whereas the lower the index the fewer the women with life-threatening conditions die (better quality of care) [10]

Among women with SMO, oxytocin was given in 3862 women (93.5 %) for prevention of postpartum hemorrhage, and only 97 of 134 women with severe PPH (72.4 %) received oxytocin as treatment. Almost all women with eclampsia (52/53; 98.1 %) received magnesium sulphate. Intra-operative prophylactic antibiotics were administered to 1409 out of 1442 women (97.7 %) who delivered by cesarean section. Only 18 of 38 women (47.4 %) with sepsis received parenteral antibiotics (Table 6).

**Table 6:** Use of interventions for prevention and treatment of major obstetric complications

Interventions	*n* (%)
Prevention of PPH	
Target population: women giving birth in health facilities	4131
Oxytocin	3862 (93.5 %)
Treatment of severe PPH	
Target population: women with severe PPH	134
Oxytocin	97 (72.4 %)
Ergometrine	105 (78.4 %)
Misoprostol	44 (32.8 %)
Other uterotonics	62 (46.3 %)
Removal of retained products	18 (13.4 %)
Artery ligation	7 (5.2 %)
Hysterectomy	4 (2.9 %)
Abdominal packing	8 (5.9 %)
Cases with SMO	52 (38.8 %)
Mortality	3 (2.2 %)
Anticonvulsants for Eclampsia	
Target population: women with eclampsia	53
Magnesium sulfate	52 (98.1 %)
Other anticonvulsant	8 (15.1 %)
Any anticonvulsant	53 (100 %)
Cases with SMO	24 (50.9 %)
Mortality	4 (7.5 %)
Prevention of cesarean-related infection	
Target population: women undergoing cesarean	1442
Prophylactic antibiotic during cesarean	1409 (97.6 %)
Treatment for sepsis	
Target population: women with sepsis	38
Parenteral therapeutic antibiotics	18 (47.4 %)
Cases with SMO	6 (15.8 %)
Mortality	2 (5.3 %)
Ruptured uterus	
Target population: women with ruptured uterus	15
Laparotomy after 3 h of hospital stay	2 (13.3 %)
Cases with SMO	12 (80.0 %)
Mortality	1 (6.7 %)

*Abbreviations*: *PPH* postpartum hemorrhage, *SMO* severe maternal outcome

## Discussion

This is the first study of SMO at a district hospital in rural Rwanda. The high prevalence of MNM in our study lies within the wide range of ratios reported in studies from other low-income countries, which used similar near miss criteria [11, 19, 20]. The high MNMR of 21.5 per 1000 live births in our study may be explained by: i) different interpretations of the criteria [12–14], ii) delayed referral of SMO cases (77.9 % compared to 20.9–64.4 % in the other studies) or iii) the high proportion of women without medical insurance which may have led to delay in seeking or providing care. The higher mortality index for MNM of 13.1 % in our and similar studies from Africa [8, 13, 14] compared to middle-income countries [5], demonstrates that a larger number of critically ill women in low-resource settings die from such severe complications.

The main direct causes of SMO were obstetric hemorrhage and hypertensive disorders, comparable to other studies in low-resource countries [11–14]. Mortality indices for these conditions were lower than in other studies, probably due to wide availability of blood for transfusion and magnesium sulfate in our setting. Particular attention is needed for conditions with a high mortality index, in particular post-cesarean sepsis and peritonitis, which were previously reported to also be a problem at a tertiary hospital in Rwanda [14]. The relative inexperience from medical officers working in district hospitals (generally recent medical school graduates) may compound this problem [21], combined with improper use of antibiotics and poor sterilization procedures [22]. These findings are extremely worrying in light of the very high cesarean section rate. This rate calls for rigorous audit of cesarean section indications [23].

Two-thirds of SMO cases were received in critical condition from other facilities or home. This may indicate difficulties in the referral system or detection of pregnant women with life threatening complications outside health facilities due to delays in seeking or reaching care [19]. Women who were referred constituted the large majority of all women with SMO. It also addresses quality of care in lower-level health facilities. When analyzing access to care, it is essential to separate near miss cases on arrival from those developing in the hospital. This would be unexpectedly high given the government’s commitment towards improving maternal and neonatal health care through use of its existing programs [17, 18] to reduce first and second delays [24]. This highlights a number of potential interventions, such as educating pregnant women and their caretakers on obstetric danger signs [21] and training health care workers at lower level facilities in emergency obstetric care [25].

MNM developed in one quarter of women with potentially life threatening conditions, with high MNMRs due to cardiovascular, renal and neurologic dysfunction. Suboptimal use of some evidence-based practices explains these SMO cases. The use of uterotonic drugs to prevent PPH was high; however, less than three out of four women who developed PPH received oxytocin as a treatment. Similarly, use of prophylactic antibiotics for cesarean delivery was high; however, less than half of women with sepsis received parenteral treatment with antibiotics. The high patient load at the facility might have a negative impact on quality of care.

The low number of women with SMO admitted into ICU is similar to what other authors in low resource settings found [25, 26]. It is easy to under-estimate severe morbidity in the absence of sophisticated laboratory diagnostics and shortage of nurse-midwives and clinicians to identify clinical signs of deteriorating patients [12, 13]. In sub-Saharan African countries, studies have shown maternal morbidity and mortality can be significantly reduced by improving maternal health care when health workers themselves use audit to identify and analyze deficiencies and apply the findings to improve their obstetric care practices [3, 27]. However, our setting is more focused on treating conditions instead of looking further on how to avoid these in the future. This type of audit will be initiated at Ruhengeri hospital as it is paramount to follow-up actions and maternal health outcomes after an audit [28].

Our study is the first study to be conducted in a rural setting in Rwanda to assess SMO using the new WHO MNM criteria. The advantage of this approach is its standardized methodology, which may allow for a comparison of health facilities and systems. Using this approach led to the integration of regular assessment of the quality of maternal health care in the hospital. In terms of limitations, the lack of follow up after discharge may lead to under reporting of actual MNM and maternal deaths. In addition, the quality of medical records was sometimes poor. We could not apply all WHO criteria due to limited resources at our facility. Some women had severe complications, which did not fulfill any clinical criteria, while laboratory criteria could not be tested for an important part. This may have caused additional under estimation of MNM. Autopsies were not performed and the underlying causes of death were only based on clinical records. Future longitudinal studies should be conducted to assess maternal morbidity and mortality using the WHO approach nationwide. Interviewing women with MNM may help to elucidate barriers to receiving hospital care.

## Conclusions

In conclusions, MNM is common at district level in Rwanda. Our study highlights some shortfalls in clinical practice and the referral system, improvements of which could lead to further reductions in maternal mortality and morbidity. We recommend intergrated continous quality of care interventions by use of audit at all levels including primary care.

## Abbreviations

CI: Confidence intervals
ICU: Intensive care unit
MI: Mortality index
MNM: Maternal Near Miss
MNMR: Maternal near miss mortality ratio
PLTC: Potentially life-threatening conditions
SMO: Severe Maternal Outcome
SMOR: Severe Maternal Outcome Ratio
WHO: World Health Organization
